# Vestibular Migraine versus Méniere’s Disease: Diagnostic Utility of Electrocochleography

**DOI:** 10.3390/audiolres13010002

**Published:** 2022-12-26

**Authors:** Paul Tabet, Ahlem Elblidi, Issam Saliba

**Affiliations:** 1 Division of Otolaryngology Head & Neck Surgery, University of Montreal Hospital Center (CHUM), Montreal, QC H2X 3E4, Canada; 2 University of Montreal Hospital Centre Research Centre (CRCHUM), Montreal, QC H2X 3E4, Canada

**Keywords:** electrocochleography, ECoG, ECochG, vestibular migraine, Méniere’s disease, migraine, anti-migraine therapy

## Abstract

Objectives: The diagnostic criteria for vestibular migraine (VM) and Méniere’s disease (MD) present an important overlap, which leads to a difficult diagnosis in patients presenting with headache, vertigo, hearing loss, ear fullness, and tinnitus. The objective of our study is to determine whether the area-under-the-curve ratio of the summating potentials (SP) and action potentials (AP) curves on electrocochleography (ECoG) helps differentiate VM from MD with or without the use of the well-established clinical criteria. Method: A retrospective review of patients filling either VM or MD criteria was undertaken between September 2015 and December 2018. All patients underwent ECoG before the introduction of anti-migraine therapy. The prediction of symptom improvement between the clinical criteria and ECoG results was compared by using the Vertigo Symptom Scale. Results: In total, 119 patients were included. An overlap of 36% exists between patients filling VM and MD criteria. Clinical criteria alone did not demonstrate a significant prediction of symptom response to anti-migraine therapy (VM 83%, MD 51%; *p* = 0.10). However, ECoG results alone did demonstrate adequate prediction (VM 94%, MD 32%; *p* < 0.001). A negative ECoG result combined with the clinical criteria of VM (100% symptom improvement) was shown to be more predictive of treatment response when compared to clinical criteria alone (83% symptom improvement) (*p* = 0.017). Finally, when used in patients filling both the VM and MD criteria (VMMD), ECoG was able to predict symptom improvement, thus better differentiating both diseases (normal ECoG: 95%, abnormal ECoG 29%; *p* < 0.001). Conclusion: Combining VM criteria with normal ECoG using the AUC ratio seems superior in predicting adequate symptom improvement than VM criteria alone.

## 1. Introduction

Vestibular migraine (VM) and Méniere’s disease (MD) are difficult to differentiate. The diagnosis of these two entities depends on specific clinical criteria. A recent systematic review conducted by our team showed an important overlap between both these sets of diagnostic criteria. In fact, we determined that of all patients that filled either the VM or MD criteria, 38% of patients met the diagnostic criteria for both [1]. Furthermore, this review highlighted mixed results when comparing VM and MD in terms of symptomatology, audiogram findings, and videonystagmography. In fact, studies have shown similar rates of aural fullness related to vertigo, pure-tone average (PTA), and caloric asymmetry between VM and MD [2,3]. Furthermore, to our knowledge, only two studies have compared the presence of endolymphatic hydrops (ELH) among MD patients and VM patients, one using magnetic resonance imaging [4] and the other using electrocochleography [3]. Both found higher rates of ELH in MD patients.

Electrocochleography (ECoG) uses acoustic stimulation of the ear to detect the presence of endolymphatic hydrops. This, in fact, has been proven in the animal model [5]. The most commonly used measure in the literature has been the amplitude ratio of the summating potentials (SP) and action potentials (AP). The results showed that the sensitivity of the SP/AP amplitude ratio was 62%. In front of this low sensitivity, ECoG is therefore seldom used in the diagnostic algorithm of VM and MD [6]. Another possible measure of ECoG is the area-under-the-curve (AUC) ratio of the SP and AP curves, with any AUC ratio above 2 being considered abnormal. Ferraro et al. were the first to report on the utility of the SP/AP area ratio in the diagnosis of MD [7]. The value of 2 as a cut-off for a normal AUC ratio is based on normative data presented in an article by Grasel et al., where the 95th populational percentile was found to be 1.67 [8]. In a recent study, Ferraro et al. determined that the use of the AUC ratio demonstrated a sensitivity as high as 96% for the diagnosis of Méniere’s disease [9,10]. Other studies, however, have shown a higher sensitivity with the SP/AP amplitude ratio for diagnosing MD when compared to the AUC ratio [11]. Nonetheless, despite these findings, ECoG as a whole is still underutilized as a diagnostic tool.

Vestibular migraine is known to respond to anti-migraine therapy such as tricyclic antidepressants, calcium channel blockers, and beta-blockers [12]. Tricyclic antidepressants (amitriptyline/nortriptyline) work by increasing extracellular serotonin neurotransmission by inhibiting its reuptake in presynaptic cells, whereas calcium channel blockers (verapamil/flunarizine) selectively inhibit the intracellular flow of calcium caused by cellular hypoxia [13,14]. On the other hand, beta-blockers (Propranolol) reduce migraine symptoms by inducing cranial vasodilatation [15].

Conversely, these medications are not considered adequate for the treatment of MD because of the absence of links between these drugs’ mechanisms and the pathophysiology of MD. Currently, our institution’s treatment algorithm for patients with an uncertain diagnosis is the following: after treatment failure with known conservative management of MD (low salt diet, diuretics), anti-migraine therapy is initiated for a minimal duration of 6 months in the presence of VM or probable VM as described by Bárány Society and the third International Classification of Headache Disorders [16]. In the advent of the failure of anti-migraine therapy, the diagnosis of VM becomes less probable, and treatment for MD (transtympanic steroid injection or surgery) is then proposed to the patient to treat potential refractory MD. We typically include endolymphatic duct blockage as a treatment alternative for MD since it has been shown to reduce symptoms of patients diagnosed with refractory MD [17].

We hypothesize that ECoG may prove to be a valuable and inoffensive tool in differentiating VM and MD without the need to uselessly treat patients with anti-migraine medication, which presents with a multitude of side effects. The objective of our study is to determine whether the amplitude ratio and AUC ratio of the SP and AP curves on ECoG help differentiate VM from MD with or without the use of well-established clinical criteria.

## 2. Method

### 2.1. Patient Selection

All patients assessed for symptoms of vertigo or dizziness between September 2015 and December 2018 by an otology and neurotology specialist (I.S.) at the University of Montreal Hospital Center (CHUM) were considered for this study. All patients underwent routine history, physical examination, and pure-tone audiometry. Patients had to meet the diagnostic criteria for vestibular migraine proposed by the Barany Society and the third International Classification of Headache Disorders [16] (Table 1) or the 2015 Amended AAO-HNS Diagnostic criteria for Meniere’s disease [18] (Table 2). If both sets of criteria were met, a “definite” diagnosis was prioritized over a “probable” diagnosis. For example, a patient filling the “definite” VM criteria and “probable” MD criteria was considered a VM patient. Inversely, a patient filling the “definite” MD criteria and “probable” VM criteria was considered an MD patient. Patients who met “definite” or “probable” criteria for both VM and MD were considered to have both diagnoses and were classified as VMMD patients.

In the presence of VM criteria, after 3 months of conservative therapy (salt restriction and diuretics), patients had to have an ECoG measuring both amplitude ratios and AUC ratios followed by a minimum of 6 months of anti-migraine therapy with either a tricyclic antidepressant (amitriptyline/nortriptyline), calcium channel blocker (verapamil/flunarizine), or beta-blocker (Propranolol). Anti-migraine therapy was dependent on individual patient comorbidities and contraindications or side effects of certain medications. If possible, patients were initially treated with the following medications in this specific order: amitriptyline or nortriptyline, verapamil, flunarizine, and propranolol. If the partial benefit was noted with the first agent, even with increased dosage, the following agent on the list was added to the first for bimodal treatment. Depending on individual patient profiles, some had unimodal treatment, while others underwent bimodal treatment. Patients with previous otologic surgery, taking vertigo-inducing medication, with a history of trauma, or with any other diagnosis of peripheral or central vertigo were excluded.

### 2.2. Variable Selection

Amplitude ratio, AUC ratio, or a combination of both were compared in order to assess which variable best correlated with the current diagnostic criteria for VM or MD. The variable showing the best correlation was then used for further analysis.

### 2.3. Study Design

All patients’ charts were retrospectively reviewed by two principal investigators (P.T. and A.E.). Vestibular symptom frequency at the initial visit was collected. Symptom improvement at 6 months following initiation of either unimodal or bimodal anti-migraine therapy was also collected. Table 3 illustrates the Vertigo Symptom Scale (VSS) Likert Scale used for the grading of symptom frequency [19]. After 6 months of anti-migraine therapy, a point drop was considered a positive response, and no change or point gain was considered a negative response. This study was approved by the University of Montreal Hospital Centre Research Centre (CRCHUM) ethics committee.

### 2.4. Electrocochleography (ECoG): Stimulus and Recording Parameters

The electrode setup and the recording parameters that we used in this study were based on the study reported by Ferraro [10]. The ground electrode was placed on the forehead (Fz), the primary electrode was placed on the eardrum, and the second electrode was placed on the contralateral mastoid. We used insert earphones delivering a broadband click with an alternating polarity at 90 dBnHL to evoke the SP and AP components.

Click stimulus was used at an intensity between 90 and 95 dBnHL with a stimulation frequency of 7 per second on a *Natus Bio-Logic® NavPRO ONE®* electrocochleography device (Middleton, WI, USA). The frequency filter range was set between 10 and 1500 Hz with a recording window of 10 msec. Juxtatympanic electrode (Lilly TM Wick, Intelligent Hearing System, Miami, FL, USA) was used when any frequency ≥ 500Hz was 60 dB or less and intratympanic electrocochleography (EMG Needle Electrode Teca^®^ Elite 28 Gauge X 25 mm Stainless Steel Sterile Concentric Needle Tip Disposable—Natus, Middleton, WI, USA) was used for any patient when any frequency ≥ 500Hz was more than 60 dB. We used 512 repetitions per trial. We obtained three repeatable trials and added the tracing before labeling the curve.

### 2.5. SP/AP Amplitude Ratio and SP/AP Area Ratio Calculation

**Figure 1:**
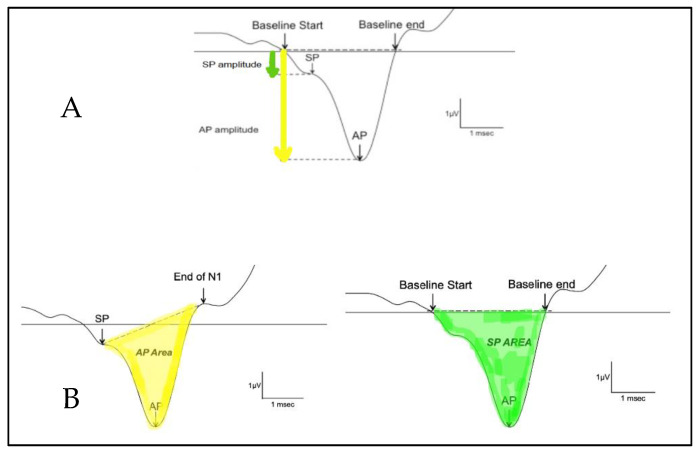
Calculation of the SP/AP ratio. (**A**) shows the amplitude and (**B**) shows the area under the curve. The AP area under the curve is defined as the area between the SP peak and the end of N1. The SP area under the curve is defined as the area between where the baseline starts and where the baseline ends.

The SP/AP amplitude ratio and SP/AP area ratio were based on Ferraro [10]. The SP and the AP amplitudes were measured from the waveform peak and compared to the pre-stimulus baseline. The SP area was defined as the area under the curve from the beginning of SP to the next point on the curve where the amplitude returned to baseline [12]. The SP/AP amplitude and area ratio was calculated using *Natus Bio-Logic® NavPRO ONE®* electrocochleography software (Figure 1). An amplitude ratio or an AUC ratio of SP and AP above 0.4 or 2, respectively, on electrocochleography was considered abnormal.

### 2.6. Data Analysis

Prediction of symptom improvement was compared between patients meeting VM criteria and patients with normal ECoG results and between patients meeting MD criteria and abnormal ECoG results. Prediction of symptom improvement between patients filling VM criteria and with normal ECoG was subsequently compared with patients filling VM criteria alone. Patients filling MD criteria and with abnormal ECoG results were also compared with patients filling MD criteria alone. Finally, we assessed whether ECoG was able to predict symptom improvement in patients filling both the VM and MD criteria (VMMD).

### 2.7. Statistical Analysis

The chi-square test and ANOVA were used to compare data from continuous variables. The chi-square test was used to compare clinical criteria to ECoG results, and logistic regression analysis was used to compare the combination of clinical criteria and ECoG results when compared to clinical criteria alone. All analyses were performed with SPSS version 22 (IBM Corp. Released 2013. IBM SPSS Statistics for Windows, Version 22.0. Armonk, NY, USA: IBM Corp).

## 3. Results

In total, 119 patients were included in this study. Of these, 41/119 (35%) were classified in the VM group, 35/119 (29%) in the MD group, and 43/119 (36%) in the VMMD group. Among the patients categorized as VM, 30/41 (73%) had a definite and 11/41 (27%) had a probable diagnosis. Among the patients categorized as MD, 22/35 (63%) had a definite and 13/35 (37%) had a probable diagnosis. Among the patients categorized as VMMD, 24/43 (56%) had a diagnosis of both definite VM and MD, and 19/43 (44%) had a diagnosis of both probable VM and MD. Amplitude ratio and the combination of amplitude and AUC ratios did not demonstrate a capability to differentiate between the VM and MD clinical criteria (*p* = 0.28; *p* = 0.24 respectively), while abnormal AUC ratio did demonstrate that capability (*p* = 0.03).

The female-to-male ratio was significantly higher in the VM group (1.68:1; *p* < 0.001) and normal ECoG group (1.70:1, *p* < 0.001) and was similar in the MD (1.58:1; *p* = 0.23) and abnormal ECoG groups (1:58; *p* = 0.08). The average age in the VM, MD, normal AUC, and abnormal AUC groups were 65.2, 62.3, 65.4, and 63.2, respectively (*p* = 0.43).

The correlation between clinical criteria and ECoG results is shown in Table 4. When comparing symptom improvement after anti-migraine treatment between the VM and MD groups, no statistically significant difference was found (VM: 83%, MD: 51%; *p* = 0.10). On the other hand, when comparing symptom improvement between patients having normal ECoG and abnormal ECoG, a statistically significant difference was found with more symptom improvement in the normal ECoG group (normal ECoG: 94%, abnormal ECoG: 32%; *p* < 0.001).

**Figure 2:**
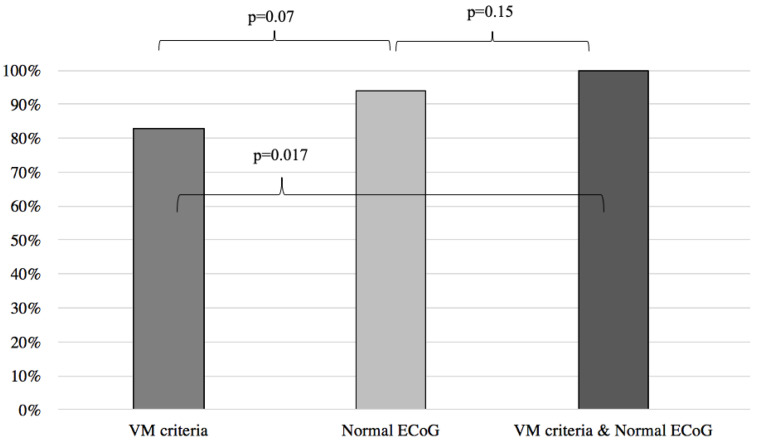
Symptom improvement comparison following anti-migraine treatment between patients with normal ECoG, positive VM criteria, and a combination of both. ECoG: electrocochleography; VM: vestibular migraine.

**Figure 3:**
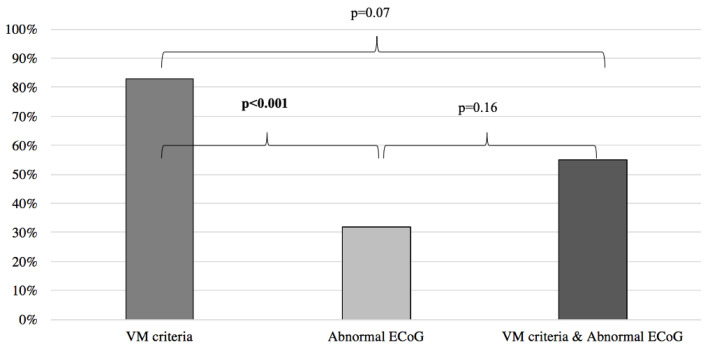
Symptom improvement comparison following anti-migraine treatment between patients with abnormal ECoG, positive VM criteria, and a combination of both. ECoG: electrocochleography; VM: vestibular migraine.

Results of symptoms improvement for the VM were then compared to the patients with normal and abnormal ECoG groups, respectively yielding similar results between the VM group and the normal ECoG group (VM: 83%, normal ECoG: 94%; *p* = 0.07) (Figure 2) and significantly better results when the VM group was compared to the abnormal ECoG group (VM: 83%, abnormal ECoG 32%; *p* < 0.001) (Figure 3). However, when the VM group was compared to a group comprised of patients filling the VM criteria and with a normal ECoG, the latter group showed a significantly better symptom improvement (VM: 83%; VM + normal ECoG: 100%; *p* = 0.017) (Figure 2). When the VM group was compared to a group comprised of patients filling the VM criteria and with an abnormal ECoG, no significant difference in symptom improvement was noted (VM: 83%; VM + abnormal ECoG: 55%; *p* = 0.07) (Figure 3).

**Figure 4:**
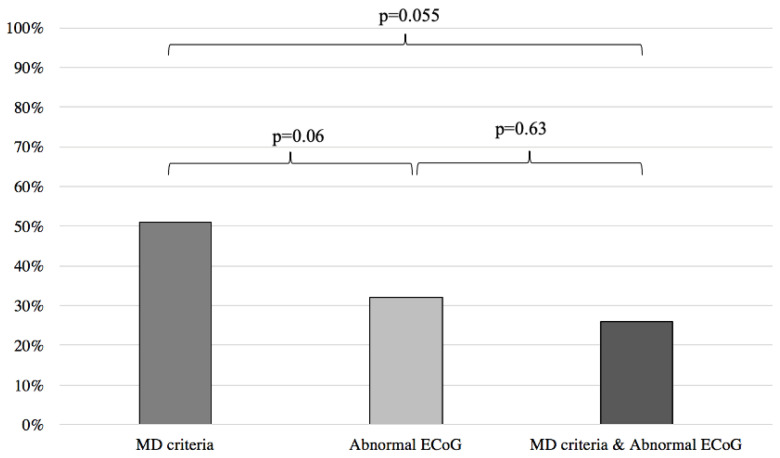
Symptom improvement comparison following anti-migraine treatment between patients with abnormal ECoG, positive MD criteria, and a combination of both. ECoG: electrocochleography; MD: Méniere’s disease.

When comparing the results of the MD and abnormal ECoG groups, we found no statistical difference between the two groups in predicting response to anti-migraine therapy (MD: 51%, abnormal ECoG: 32%; *p* = 0.06) (Figure 4).

**Figure 5:**
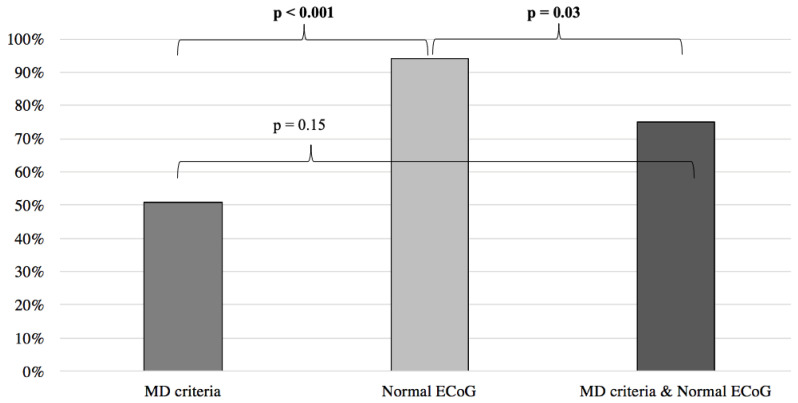
Symptom improvement comparison following anti-migraine treatment between patients with normal ECoG, positive MD criteria, and a combination of both. ECoG: electrocochleography; MD: Méniere’s disease.

Inversely, when compared to the MD clinical criteria, normal ECoG proved to be a better predictor of symptom improvement following anti-migraine therapy (MD: 51%; normal EcoG: 94%; *p* < 0.001) (Figure 5). Furthermore, no difference was noted when comparing the MD group with patients filling the MD criteria and presenting an abnormal ECoG (MD: 51%, MD + abnormal ECoG 26%; *p* = 0.055) (Figure 4). When the MD group was compared to patients filling the MD criteria and presenting normal ECoG, no significant difference in symptom improvement was noted (MD: 51%; MD + normal ECoG: 75%; *p* = 0.15) (Figure 5).

Finally, when used in patients filling both the VM and MD criteria (VMMD), ECoG was able to predict symptom improvement, thus better differentiating both diseases (normal ECoG: 95%, abnormal ECoG 29%; *p* < 0.001).

## 4. Discussion

In our previous systematic review of patients filling VM and MD criteria, an overlap of 38% was found [1]. This result is like the one found in our series (36%). Possible disparities in our VM and MD prevalence, when compared to the literature, may be explained by the lack of a VMMD group in many studies, as stated in our previous 2017 systematic review [1]. Another possible explanation for this may be the recent changes in diagnostic criteria for VM (2012) and MD (2015). Normal ECoG was more frequently found in the VM group, and abnormal ECoG was more prevalent in the MD group (Table 4). These results are similar to ones found in the literature, which also showed a higher prevalence of endolymphatic hydrops in MD patients when compared to VM patients [3,4].

According to our results, clinical criteria alone are insufficient in predicting symptom improvement. This reinforces the argument that the clinical criteria should not be used alone to guide the management of these patients. On the other hand, the normal AUC ratio on ECoG seems sufficient for adequate prediction. However, when compared to clinical criteria, normal ECoG only trends towards being better at identifying those responding to anti-migraine therapy. Therefore, given the simplicity and necessity of history taking in these patients, clinical criteria should not be overlooked. In fact, the combination of VM criteria and normal ECoG showed an even higher rate of adequate symptom improvement prediction when compared to ECoG results alone (94% vs. 100%). However, the combination of MD criteria and abnormal ECoG did not show a better prediction of negative response to anti-migraine therapy when compared to MD criteria alone. In a recent study, Hornibrook et al. demonstrated that the use of tone-burst in ECoG led to a better sensitivity in diagnosing Méniere’s disease [20]. We hypothesize that the use of tone bursts instead of clicks may help to improve the sensitivity of ECoG for the prediction of symptom improvement. A future prospective study using tone-burst ECoG may, in fact, show that ECoG is a significantly better predictor of symptom improvement than both the VM and MD criteria.

**Figure 6:**
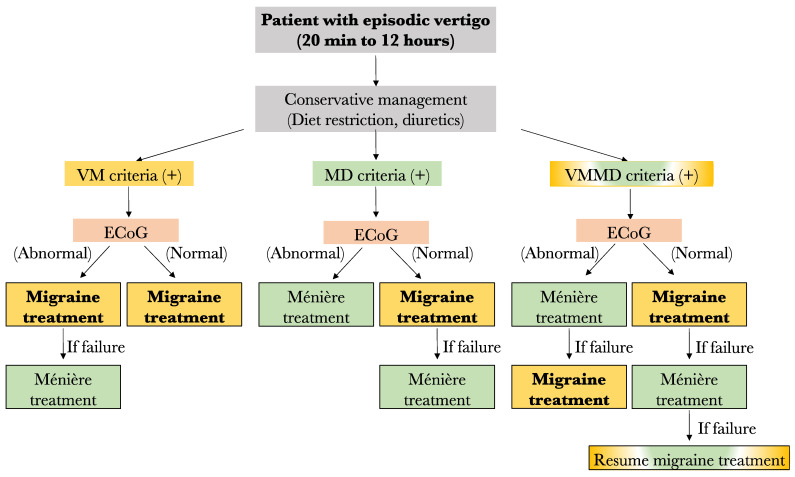
Proposed algorithm for the management of patients with episode vertigo. VM: vestibular migraine; MD: Méniere’s disease; ECoG: electrocochleography (Yellow fr VM, Green for MD, yellow and green for both diseases).

For now, the results of this study lead us to propose the following preliminary treatment algorithm in case of no response to low salt diet and diuretics: (1) Any patient filling only the VM criteria should be started on anti-migraine therapy for 6 months. However, if an abnormal ECoG is obtained prior to treatment and no response to anti-migraine therapy is noted, a treatment for MD should be considered. (2) Among patients filling only the MD criteria, those with an abnormal ECoG result may be offered specific MD treatment such as transtympanic injections, oral steroids, or surgery, depending on patient preference and severity of symptoms. In fact, given the low response rate to anti-migraine therapy in this group (63%), MD therapy may be considered before VM treatment. On the other hand, those with normal ECoG results should be started on anti-migraine therapy prior to considering MD treatment. (3) Similarly, the patients filling both the VM and MD criteria should have their treatment in accordance with ECoG results. Patients with normal ECoG should be offered anti-migraine therapy first, and those with abnormal ECoG should be offered MD treatment prior to anti-migraine therapy. A summary of the proposed treatment algorithm is shown in Figure 6.

## 5. Limitations

This pilot study is primarily limited by its low power. Insufficient sample size may, in fact, lead to possible type II errors. However, all significant differences found remain interesting findings, which should motivate physicians to further consider ECOG as a useful diagnostic tool. Additionally, given the small sample size and already low power, subgroup analysis for confounding data such as individual specific anti-migraine therapy and vestibular suppressant use was not carried out. The creation of a control group among both VM and MD groups would have been ideal to clearly assess the efficacy of treatment as opposed to the natural evolution of the disease. Data here were collected retrospectively after initiation of treatment, and all patients were, in fact, treated by some form of medication, making the creation of a control group impossible.

Despite the attractiveness of the AUC SP/AP ratio, there is no consensus in the literature on how to calculate the SP and AP areas. It is advisable that each clinician interpreting ECoG should understand how the used evoked potential system calculates SP/AP amplitude and area ratio. Each Evoked Potential system manufacturer should include an explanation of the software formula used for the calculation of SP/AP amplitude and area ratio.

Furthermore, history taking for these patients was conducted without the use of a validated vertigo questionnaire, such as the vertigo symptom scale (VSS) or dizziness handicap index (DHI). However, the VSS Likert scale is a proven method to quantify the frequency of vertigo/dizziness symptoms [17]. Additionally, anti-migraine therapy differed between patients, given their individual contraindications and side effect profiles. An ideal study would have used one anti-migraine agent for all patients, excluding those unable to tolerate therapy. Given the limited number of patients included in this study, patients with different regimens were included.

Even though our previous review did not find any vestibular test able to differentiate VM from MD, we are completing for all our patients the caloric testing, the vestibular evoked myogenic test (VEMP), and the video head impulse test as well [1]. During the physical exam, video-Frenzel findings and skull vibration test could be a part of the evaluation, but still, no data in the literature show the advantages of these tools to differentiate VM from MD. The same conclusion is considered concerning the 3 Tesla magnetic resonance imaging.

For all the reasons cited above, we emphasize that this paper must be considered a pilot study and that the aforementioned treatment algorithm must be seen only as a preliminary suggestion based on our initial findings. A prospective study is already underway at our institution, incorporating a more complete assessment tool and sufficient study power.

## 6. Conclusions

Firstly, an overlap of 36% exists between patients filling VM and MD criteria, and these criteria alone do not adequately predict treatment response to anti-migraine therapy. Our data find that combining VM criteria with normal ECoG using the AUC ratio seems superior to predicting adequate symptom improvement than VM criteria alone.

## Figures and Tables

**Table 1:** Diagnostic criteria for vestibular migraine proposed by Bárány Society and the third International Classification of Headache Disorders (ICHD-3) [16].

	1. Vestibular Migraine
A	At least 5 episodes with vestibular symptoms of moderate or severe intensity, lasting 5 min to 72 h
B	Current or previous history of migraine with or without aura according to the International Classification of Headache Disorders (ICHD)
C	One or more migraine features with at least 50% of the vestibular episodes:
	(1) Headache with at least two of the following characteristics: One-sided location Pulsating quality Moderate or severe pain intensity Aggravation of routine physical activity
	(2) Photophobia and phonophobia
	(3) Visual aura
D	Not better accounted for by another vestibular or ICHD diagnosis
	**2. Probable vestibular migraine**
A	At least 5 episodes with vestibular symptoms of moderate or severe intensity, lasting 5 min to 72 h
B	Only one of the criteria B and C for vestibular migraine is fulfilled (migraine history or migraine features during the episode)
C	Not better accounted for by another vestibular or ICHD diagnosis

**Table 2:** Amended 2015 Criteria for Diagnosis of MD [18].

**Definite**	Two or more spontaneous episodes of vertigo, each lasting 20 min to 12 h
	Audiometrically documented low-to mid-frequency sensorineural hearing loss in 1 ear, defining the ear on 1 occasion before, during, or after 1 episode of vertigo
	Fluctuating aural symptoms (hearing, tinnitus, or fullness) in the affected ear
	Not better accounted for by another vestibular diagnosis
**Probable**	Two or more episodes of vertigo or dizziness, each lasting 20 min to 24 h
	Fluctuating aural symptoms (hearing, tinnitus, or fullness) in the affected ear
	Not better accounted for by another vestibular diagnosis

**Table 3:** Vertigo symptom scale Likert Scale [19].

Points	Frequency of Vertigo or Dizziness Symptoms
0	Never
1	1–3 times a year
2	4–12 times a year
3	>1 per month
4	>1 per week

**Table 4:** Clinical criteria versus ECoG SP/AP AUC ratio for prediction of vestibular symptom improvement.

	Normal ECoG	Abnormal ECoG	*p*-Value
VM group	32/41 (78%)	9/41 (22%)	<0.001
MD group	13/35 (37%)	22/35 (63%)	0.03
VMMD group	21/43 (49%)	22/43 (51%)	0.82

ECoG: electrocochleography; SP: summating potential; AP: action potential; AUC: area under the curve; VM: vestibular migraine; MD: Méniere’s disease; VMMD: combined vestibular migraine and Méniere’s disease.

## Data Availability

Data available on request due to restrictions (ethical).
